# Challenges and Strategies for Breeding Resistance in *Capsicum annuum* to the Multifarious Pathogen, *Phytophthora capsici*

**DOI:** 10.3389/fpls.2018.00628

**Published:** 2018-05-15

**Authors:** Derek W. Barchenger, Kurt H. Lamour, Paul W. Bosland

**Affiliations:** ^1^Department of Plant and Environmental Sciences, New Mexico State University, Las Cruces, NM, United States; ^2^Department of Entomology and Plant Pathology, The University of Tennessee, Knoxville, Knoxville, TN, United States

**Keywords:** pepper, root-rot, stem-blight, fruit-blight, oomycete

## Abstract

*Phytophthora capsici* is the most devastating pathogen for chile pepper production worldwide and current management strategies are not effective. The population structure of the pathogen is highly variable and few sources of widely applicable host resistance have been identified. Recent genomic advancements in the host and the pathogen provide important insights into the difficulties reported by epidemiological and physiological studies published over the past century. This review highlights important challenges unique to this complex pathosystem and suggests strategies for resistance breeding to help limit losses associated with *P*. *capsici*.

## Introduction

The soil-borne oomycete plant pathogen *Phytophthora capsici* (Leon.) is the most devastating pathogen to chile pepper production. Chile pepper is an increasingly important crop used as a vegetable, spice, food colorant, and medicinal applications. Over the last 30 years, chile pepper consumption has increased 40-fold (Rehrig et al., 2014). Chile pepper is a high-value crop and has immediate economic benefits for producers. Additionally, chile peppers are important sources of essential nutrients providing long term nutritional benefits for consumers. Globally, *P*. *capsici* causes more than $100 million in losses annually (Bosland, 2008). This enormity of damage has stimulated extensive collaborations between plant pathologists and plant breeders to better understand the epidemiology of the pathogen as well as the mechanisms of resistance in the host. This review highlights recent work with the *Capsicum*–*Phytophthora* pathosystem and discusses novel approaches to more effectively manage this devastating disease.

### Pathogen Identification

*Phytophthora capsici* was first reported in New Mexico by Fabián García as a “souring of the soil” (García, 1908). In 1922, Leonian systematically described *P*. *capsici* isolated from chile pepper in 1918 at the New Mexico Agricultural Research Station in Las Cruces, NM, United States (Leonian, 1922). Synonyms for *P*. *capsici* include *P*. *hydrophila* (Cruzi, 1927), *P*. *parasitica* var. *capsici* (Sarejanni, 1936), and *P*. *palmivora* MF4 (Griffin, 1977).

*Phytophthora capsici* is part of a species complex with several attempts at resolution over the years, but it has not been fully determined whether it is one species with *formae speciales*, or multiple species. Studies of *P*. *capsici* populations recovered from vegetables at diverse locations reveal a very high level of heterozygosity, typical for an obligately outcrossing diploid organism. Despite individual isolates carrying a large complement of genetic variation, populations can vary dramatically, with some comprised almost entirely of long-lived clonal lineages (e.g., Peru, Argentina, Taiwan, and portions of China) and others displaying a wide array of diverse genotypes that change yearly due to the requirement for sexual recombination and development of thick-walled oospores to survive (e.g., United States and Mexico) (Gobena et al., 2012b; Hu et al., 2013a,b; Castro-Rocha et al., 2016, 2017; Barchenger et al., 2017). The extensive of genetic variation may play a part in attacking such a large host group. Satour and Butler (1967) found 45 species of cultivated plants and weeds susceptible to *P. capsici*. There is currently a lack of clear delineation between *P*. *capsici*, the closely related *P*. *tropicalis* and multiple, un-named, but clearly evolutionarily distinct species (Lamour et al., 2012). This is due to the historical use of spore shape (sporangial length/breadth ratio), pedicel length and caducity, and the amphigynous oospore structure – which are poor characters for defining evolutionary relationships, and the difficulty in knowing how much genetic differentiation is sufficient to fully resolve the species. In practice, isolates recovered from woody or perennial hosts are not the same species as isolates recovered from herbaceous annual plants (Lamour et al., 2012).

### Disease Symptoms

*Phytophthora capsici* causes root-rot as well as stem-, leaf-, and fruit-blight. These disease syndromes are dependent on host species, point of infection, and also are influenced by environmental conditions. Furthermore, disease severity is affected by plant maturity, with more mature plants generally being more resistant than seedlings or young fruit (Erwin and Ribeiro, 1996; Lamour et al., 2011; Mansfeld et al., 2017).

In chile pepper, the root-rot syndrome caused by *P*. *capsici* is associated with root darkening and small lesions that can quickly expand to girdle and kill the root. In seedlings, damping off associated with root-rot can kill plants two to 5 days after inoculation (Erwin and Ribeiro, 1996). In older plants, root infections can result in stunting, wilting, and eventual plant death in approximately 2 weeks. Root-rot is the most destructive and economically important disease syndrome of chile pepper (Walker and Bosland, 1999; Bosland, 2008).

Foliar-blight symptoms include dark, water soaked areas of the leaves (Walker and Bosland, 1999). The disease starts with a small circular or irregular-shaped lesion on the leaves giving a “scalded” appearance. Later, the lesions enlarge, dry, and bleach to a light tan. The disease progresses to the stem as a dark-green and water-soaked lesion. Finally, the plant is defoliated and stems dry and brown (Weber, 1932). Infected leaves will turn brown or tan and may defoliate as infection spreads to the stem (Alcantara and Bosland, 1994). Stem-blight and crown-rot symptoms of chile pepper are often similar. These symptoms include distinctive black or purple lesions near the soil line (Erwin and Ribeiro, 1996; Ristaino and Johnston, 1999). The lesions rapidly coalesce and girdle the main branches of stem, which results in stem or entire plant death (Erwin and Ribeiro, 1996).

The early symptoms of fruit-blight include small, water-soaked, dull-colored spots that can rapidly elongate under favorable conditions. Fruit-blight symptoms can continue to spread until most of the chile pepper pod is symptomatic, resulting in unmarketable fruit. Lesions generally occur at either the stem end or the blossom tip of the fruit, but can spread quickly toward the center of the fruit (Erwin and Ribeiro, 1996). The infected tissue becomes dry, sunken, and paper-like and will often turn a tan or straw color.

### Management Strategies

Phytophthora blight encompasses both below-ground and above-ground symptoms (Leonian, 1922). Conditions conducive to root infection by *P*. *capsici* are saturated soil for extended periods and warm soil temperatures (Weber, 1932; Walker and Bosland, 1999). Free water in the soil from rainfall and irrigation has a greater effect on disease severity than the initial concentration of inoculum (Ristaino, 1991). Additionally, *Phytophthora* outbreaks may be more severe in low or shaded areas of a field, due to slow drying in these areas (Bosland and Lindsey, 1991; Goldberg, 1995; Hausbeck and Lamour, 2004).

Foliar-blight and stem-blight are serious problems in areas with high relative humidity (Gevens et al., 2007) or during the fall rainy period in other regions (Barksdale et al., 1984; Alcantara and Bosland, 1994). Splashing water due to heavy rainfall or overhead irrigation may allow normally soil-borne *P. capsici* to infect aerial plant parts (Black, 1999). The disease may also result from sporangia and zoospores produced on diseased plant parts when environmental conditions are favorable. In New Mexico, United States, plants are contaminated when fruit pickers spread infested soils onto wet leaves; especially when harvesting early in the morning when dew is on the leaves.

Current management practices for *Phytophthora* are cultural, chemical and planting resistant hosts. These approaches include irrigation management, crop rotation, soil solarization, fungicide applications (Ristaino and Johnston, 1999; Sanogo, 2003; Hausbeck and Lamour, 2004; Granke et al., 2012; Sanogo and Bosland, 2012), and the planting of cultivars that are resistant to local isolates. Generally, these management strategies aim to limit losses associated with the pathogen because once established, *P*. *capsici* is very difficult to eradicate (Lamour et al., 2011). Additionally, *P*. *capsici* can readily move from field to field and rapidly establish itself in a given region, as surface water used for irrigation is an important means of disseminating the pathogen (Gevens et al., 2007). Extreme weather events (e.g., flooding, hurricanes, or typhoons) can initiate new and widespread infestations (Sheu et al., 2009; Dunn et al., 2010). Since fully restricting the movement of *P*. *capsici* among sites is often impossible, the best approach to prevent *P*. *capsici* infection in vegetable crops is the development of resistant cultivars because it is less expensive and a sustainable alternative to fungicide applications and other management practices (Hausbeck and Lamour, 2004).

### Host Range

Originally considered to be host specific (Tucker, 1931), it has since been shown that *P*. *capsici* can infect many other plant species including cultivated crops, ornamentals, and native plants belonging to more than 15 plant families (Satour and Butler, 1967; Erwin and Ribeiro, 1996; Hausbeck and Lamour, 2004; Tian and Babadoost, 2004; French-Monar et al., 2006; Granke et al., 2012). It is a major threat to the important crop plant families Cucurbitaceae, Fabaceae, and Solanaceae (Hausbeck and Lamour, 2004). Soon after its identification in 1922, *P*. *capsici* was reported to infect eggplant (*Solanum melongena* L.) (Cruzi, 1927). *Phytophthora capsici* was first reported to infect cucurbits when Kreutzer (1937) isolated *P*. *capsici* in a field of cucumber (*Cucumis sativus* L.). Three years later, the pathogen was reported to infect muskmelon (*C*. *melo* L.), summer squash (*Cucurbita pepo* L.), and tomato (*S*. *lycopersicum* L.) (Kreutzer et al., 1940; Wiant and Tucker, 1940).

In addition to members of the families Cucurbitaceae, Fabaceae, and Solanaceae, Satour and Butler (1967) found the annual crops of okra (*Abelmoschus esculentus* L.), safflower (*Carthamus tinctorius* L.), and spinach (*Chenopodium amaranticolor* Coste and Reyn.) as well as onion (*Allium cepa* L.) (Leu and Kao, 1981) are hosts of *P. capsici*. Additionally, woody perennial crops such as apple (*Malus pumila* Mill.) (Wiant and Tucker, 1940), avocado (*Persea americana* Mill.) (Tompkins and Tucker, 1937), black pepper (*Piper nigrum* L.) (Tsao et al., 1985), cacao (*Theobroma cacao* L.) (Zentmyer et al., 1977), fig (*Ficus carica* L.) (Katsura and Tokura, 1955), Fraser fir (*Abies fraseri* Pursh.) (Quesada-Ocampo et al., 2009), macadamia (*Macadamia integrifolia* Maiden & Betche) (Hunter et al., 1971), papaya (*Carica papaya* L.) (Erwin and Ribeiro, 1996), peach (*Prunus persica* L.) (Tompkins and Tucker, 1937), and rubber (*Hevea brasiliensis* Müll. Arg.) (Erwin and Ribeiro, 1996) are also host species. Most of these isolates likely belong to evolutionarily distinct species and can no longer share genetic information via mating with the vegetable strains (Donahoo and Lamour, 2008).

### Pathogen Distribution

After its first identification, *P*. *capsici* was quickly recognized throughout important production regions in the United States. Following New Mexico, the pathogen was identified in California (Tompkins and Tucker, 1937, 1941) in the 1920s, in Colorado (Bodine, 1935; Sandsten, 1939), Florida (Weber, 1932), Arizona (Brown and Evans, 1933), and New York (Wiant and Tucker, 1940) in the 1930s, Texas in the 1940s (French-Monar et al., 2009, New Jersey in the 1960s (Barksdale et al., 1984; Parra and Ristaino, 1998), Hawaii in the 1970s (Hunter et al., 1971), and in South Carolina (Quesada-Ocampo et al., 2011), Michigan (Hausbeck and Lamour, 2004), and Illinois (Babadoost, 2000) in the 1990s. Today, *P. capsici* is likely established in every state (Hausbeck and Lamour, 2004; Quesada-Ocampo et al., 2011; Granke et al., 2012).

**FIGURE 1:**
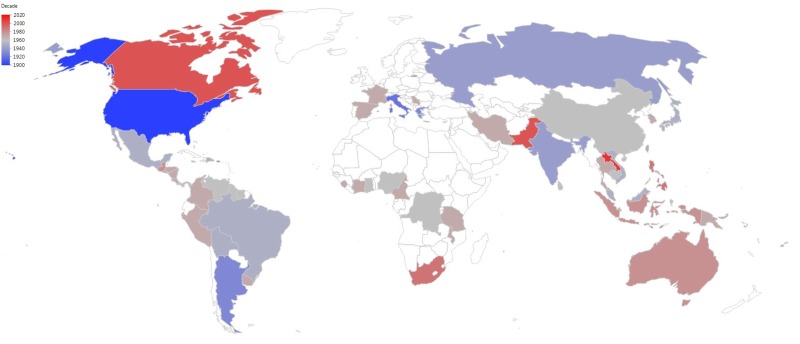
Progressive global spread of *Phytophthora capsici* from first identification in New Mexico, United States in the early 1900s through to 2017. The countries that are white in color may have *P*. *capsici*; however, no reports identifying the pathogen have been published.

Although it is not known how the pathogen is spread over long distances (Lamour et al., 2011), *P. capsici* is truly a global disease (Cruzi, 1927; Tucker, 1928; Sarejanni, 1936; Marchionatto, 1938; Godoy, 1940; Thomas et al., 1947; Osnitzkaya, 1949; Malaguti and Pontis-Videla, 1950a,b; Do Amaral, 1952; Katsura and Tokura, 1954; Bell and Alandia, 1957; Turner, 1960, 1961a,b; Holliday and Mowat, 1963; Ravise, 1966; Brasier, 1969; Ershad, 1971; Fernandez-Northcote, 1971; Alfaro Moreno and Vegh, 1972; Clerjeau, 1973; Zentmyer et al., 1973; Aleksić et al., 1975; Kim et al., 1975; Griffin, 1977; Tsao and Tummakate, 1977; Zhou et al., 1984; Alizadeh and Tsao, 1985; Tsao et al., 1985; Carter, 1986; Mu and Tsao, 1987; Tsao and Mu, 1987; Romero-Cova, 1988; Ho, 1990; Thompson et al., 1994; Anderson and Garton, 2000; Gilbert et al., 2001; Guigón-López and González-González, 2001; Velásquez-Valle et al., 2001; Pérez-Moreno et al., 2003; Noveriza and Quimio, 2004; Silvar et al., 2006; Sholberg et al., 2007; Silva-Rojas et al., 2009; Vásquez-López et al., 2009; Zapata-Vázquez et al., 2012; Nguyen, 2015; Callaghan et al., 2016). The chronological spread of *P*. *capsici* is presented in **Figure 1**. However, this is likely not an exhaustive list, as the pathogen could be present in other countries and just not yet reported.

## Challenges

For nearly a century, researchers around the world have studied the *Capsicum*–*Phytophthora* pathosystem, making great strides in understanding this complex interaction. However, even with the greater knowledge gained, the global incidence of the disease is increasing (Hwang and Kim, 1995; Ristaino and Johnston, 1999; Parra and Ristaino, 2001; Kousik and Keinath, 2008; Stam et al., 2013) and most commercial cultivars are either very susceptible or only partially resistant to *P*. *capsici* (Ristaino and Johnston, 1999; Hausbeck and Lamour, 2004; Café-Filho and Ristaino, 2008). Progress is slow in limiting losses associated with *P*. *capsici* because of the unique challenges presented by this devastating pathogen.

### Fungicide Resistance

Although morphologically similar to fungi, oomycetes are genetically and biochemically divergent (Erwin and Ribeiro, 1996) and are generally not sensitive to most broad-spectrum fungicides (Davidse et al., 1991). Therefore, the fungicides growers can rely on to manage oomycetes are limited (Lamour and Hausbeck, 2000). Metalaxyl (Ridomil^®^; Syngenta) is a phenylalamide fungicide introduced in 1977 that provided systemic protection against oomycetes diseases, including *Phytophthora* sp. (Cohen et al., 1979; Davidse, 1995; Schwinn and Staub, 1995). Metalaxyl has been used to manage root- and crown-rot of chile pepper (Papavizas and Bowers, 1981; Johnston, 1982; Schlub and Johnston, 1982; Hwang and Sung, 1989; Ristaino et al., 1993; Matheron and Matejka, 1995). Metalaxyl was replaced with mefenoxam (Ridomil Gold^®^; Syngenta), contains the active enantiomer contained in metalaxyl (Parra and Ristaino, 2001) and has been widely used to manage *P*. *capsici* (Lamour and Hausbeck, 2000; Silvar et al., 2006).

The mode of action of phenylamide fungicides is site specific, and fungicide insensitivity was observed in susceptible plant pathogens soon after their introduction in the 1970s (Lamour and Hausbeck, 2000). Insensitivity to mefenoxam and metalaxyl has been widely observed in *P. capsici* (Bruin and Edington, 1981, 1982; Bower and Coffey, 1985; Abdellaoui-Maane et al., 1988; Lucas et al., 1990; Miller et al., 1994; Hwang and Kim, 1995; Parra and Ristaino, 1998, 2001; Mathis et al., 1999; Ristaino and Johnston, 1999; Agosteo et al., 2000; Lamour and Hausbeck, 2000, 2001b, 2003; Louws et al., 2000; Stevenson et al., 2000, 2001; Babadoost and Islam, 2001; Ploetz R.C. et al., 2001; Tamietti and Valentino, 2001; Matheron and Porchas, 2002; McGovern et al., 2003; Seebold and Horten, 2003; Zhang and Liang, 2003; Hausbeck and Lamour, 2004; McGrath, 2004; Waldenmaier, 2004; French-Monar et al., 2006; Silvar et al., 2006; Gevens et al., 2007; Café-Filho and Ristaino, 2008; Qi et al., 2008; Liu et al., 2009).

Given the global emergence of insensitivity to phenylamide fungicides in *P. capsici*, alternative fungicides have been evaluated (Cui et al., 2009; Sun et al., 2010; Bi et al., 2014). Some of these compounds include azoxystrobin, cyazofamid, cymoxanil, dimethomorph, fluazinam, fosetyl-A1, oligochitosn, oxathiapiprolin, and zoxmide (Matheron and Porchas, 2000; Ivors et al., 2006; Keinath, 2007; Xu et al., 2007a,b; Ji and Csinos, 2015). However, soon after their first use to manage the disease, insensitivity is often observed (Kousik and Keinath, 2008; Cui et al., 2009; Sun et al., 2010; Bi et al., 2014; Miao et al., 2016).

The unusually rapid and high preponderance of fungicide insensitivity in *P*. *capsici* is likely due to the pathogen’s ability to sexually reproduce resulting in high rates of genetic recombination in addition to the production of oospores that can persist in the soil for many years (Lamour and Hausbeck, 2000, 2001b; Bi et al., 2014). Additionally, this is further evidence that resistant cultivars are the best management strategy for *P*. *capsici*. In *Phytophthora*, insensitivity to the phenylamide class of fungicides has been reported to be controlled by a single major effect locus with incomplete dominance that is subject to modifying genes with minor effects (Shattock, 1988; Chang and Ko, 1990; Bhat et al., 1993; Fabritius et al., 1997; Lamour and Hausbeck, 2000). Additionally, once mefenoxam insensitivity has been introduced into a population it is persistent and the frequency of insensitive individuals does not decrease after selection pressure is removed (Bower and Coffey, 1985; Lamour and Hausbeck, 2001b). Sensitivity to dimethomorph was found to be controlled by two dominant genes (Bi et al., 2014) and oxathiapiprlin by a single gene (Miao et al., 2016).

### Multiple Disease Syndromes

As previously stated, depending on the point of infection, growing environment, and plant maturity, *P*. *capsici* can cause disease on effectively every part of the chile pepper plant (Alcantara and Bosland, 1994; Goldberg, 1995; Ristaino and Johnston, 1999; Walker and Bosland, 1999; Sy et al., 2005). For each *P. capsici* disease syndrome (root-rot, foliar-blight, stem-blight, and fruit-blight) separate and independent resistant systems have evolved in the host (Monroy-Barbosa and Bosland, 2010), requiring the presence of independent resistance genes for the control of each disease syndrome (Walker and Bosland, 1999; Sy et al., 2005).

The necessity of independent resistance genes for each of the multiple disease syndromes caused by *P*. *capsici* in chile pepper increases the complexity of resistance breeding. For host resistance, plant breeders have to pyramid multiple resistance genes in a cultivar to a single race of *P*. *capsici*. A similar phenomenon has been observed in the closely related pathosystem of potato (*S*. *tuberosum* L.) and *P*. *infestans* ([Mont.] de Bary) (Bonde et al., 1940; Rudorf et al., 1950).

### Multitude of Races

Within the Phytophthora root-rot and foliar-blight disease syndromes, more than 45 physiological races for have been identified (Hwang et al., 1995; Oelke et al., 2003; Glosier et al., 2008; Sy et al., 2008; Lee et al., 2010; Monroy-Barbosa and Bosland, 2011; da Costa Ribeiro and Bosland, 2012; Jiang et al., 2015; Barchenger, 2017) with different *R* genes controlling the resistant phenotype against each physiological race of *P*. *capsici* within each disease syndrome (Monroy-Barbosa and Bosland, 2008). Screening for resistance has been accomplished on a wide range of genetic material (Kimble and Grogan, 1960; Barksdale et al., 1984; Peter et al., 1984; Reifschneider et al., 1986; Ortega et al., 1991; Candole et al., 2010), and sources for *P*. *capsici* resistance have been identified in *C*. *annuum* such as Criollo de Morelos 334 (CM334), PI 201232, PI 201234, PI 201237, and PI 640532 (McGregor et al., 2011) from southern Mexico, AC2258 from Central America, and ‘Perennial’ from India. Among the sources of resistance, CM334 has the highest resistance level (Quirin et al., 2005). It is proposed that the center of origin for *P*. *capsici* is Mexico, Central or South America (Zentmyer, 1988). The reason the majority of *P*. *capsici* resistant chile peppers are from this region can be explained by an evolutionary arms race. This coevolution results in plant specificity and pathogen virulence continually adapting in response to each other. For this reason, breeding for *P*. *capsici* resistance in chile pepper is challenging, because new races are continually evolving to overcome the host resistance.

Several *P*. *capsici* race identification systems have been proposed (Black, 1999; Oelke et al., 2003; Glosier et al., 2008; Lee et al., 2010); however, these relied on the use of cultivars as the host differential. Using chile pepper cultivars for race detection has limitations because cultivars can vary among seed companies and can segregate (Votava and Bosland, 2002; Candole et al., 2012). Additionally, cultivars can become unavailable, and not all accessions are available to scientists in different countries. Sy et al. (2008) developed a differential set of New Mexico Recombinant Inbred Lines (NMRIL) for *P*. *capsici* race characterization that been used for large scale race detection. Recombinant inbred lines (RILs) are often used as host differentials to identify races of pathogens (Lister and Dean, 1993). The RILs allow the maximum genetic variability within a population with homozygous genotypes that can be replicated permanently without the risk of segregation occurring. The NMRILs have been used for race detection in the United States (Monroy-Barbosa and Bosland, 2008, 2010, 2011; Sy et al., 2008; Jiang et al., 2015), Brazil (da Costa Ribeiro and Bosland, 2012), and Taiwan (Barchenger, 2017). The NMRILs have the potential to differentiate thousands of races of *P*. *capsici* based on the formula 2^n^, where 2 is the number of possible reactions (resistant and susceptible) and n is the number of host differentials used.

### Mating Type and Genetic Recombination

*Phytophthora capsici* is a heterothallic species that can reproduce both asexually and sexually (Erwin and Ribeiro, 1996). Once the pathogen is introduced into a field and exposed to water (such as rainfall or irrigation), *P*. *capsici* rapidly reproduces asexually through the production of sporangia and motile zoospores (Hausbeck and Lamour, 2004; Lamour and Kamoun, 2009). Each sporangium can produce 20–40 zoospores that can travel in standing water and infect nearby plants (Hausbeck and Lamour, 2004). This swift spread throughout a field can result in losses up to 100% within days. For isolates recovered from the middle and eastern United States, there is no evidence to suggest host specialization (Castro-Rocha et al., 2017). Infection on a single cucumber or pumpkin easily leads to 100’s of millions of spores being released during a rain or irrigation event. Recent studies indicate zoospore progeny can have genomes markedly different, at the chromosome level, from the isolate (or isolates) that initiated the infection and that an impressive, and potentially highly significant, amount of asexual evolution is occurring during spore production (Barchenger et al., 2017; Castro-Rocha et al., 2017; Shrestha et al., 2017).

Sexual reproduction occurs when the two mating types that have been designated as A1 and A2 (Erwin and Ribeiro, 1996), are in close proximity. Exposure to mating type specific hormones α1 and α2 stimulates production of gametangia, outcrossing, and recombinant oospore formation (Ko, 1988). Interestingly, both mating types also produce male and female gametangia and are capable of self-fertilization (Ko, 1988). However, self-fertilization is not likely to occur as often as outcrossing (Uchida and Aragaki, 1980; Dunn et al., 2014a). These recombinant oospores can survive extended periods of cold temperatures (Hausbeck and Lamour, 2004; Babadoost and Pavon, 2013) and are the source of overwintering inoculum in regions with cold winter conditions (Bowers, 1990; Lamour and Hausbeck, 2003; Granke et al., 2012). Regardless of host availability, oospores have been observed to remain viable in diverse soil textures for several years (Babadoost and Pavon, 2009). When the oospores are exposed to a susceptible host and favorable conditions, they rapidly initiate the repeating asexual reproductive cycle and begin their progression throughout the field (Hausbeck and Lamour, 2004; Granke et al., 2012).

Recently, Carlson et al. (2017) identified a 1.6 Mbp region associated with mating type determination, designated the “mating type region” (MTR) in a closed bi-parental field population in New York, United States. This population started with diploid parents and the authors report elevated heterozygosity across the MTR for the A2 mating type relative to the A1 mating type followed intensive inbreeding. This finding was supported by Barchenger et al. (2017), who found that the A1 isolates collected in Taiwan were largely diploid and the A2 isolates were generally triploid or higher ploidy.

Both A1 and A2 mating types of *P*. *capsici* have been widely identified within the same field (Ristaino, 1990; Pan, 1997; Parra and Ristaino, 1998, 2001; Lamour and Hausbeck, 2000, 2001a, 2002; Ploetz R. et al., 2001; Fernandez-Pavia et al., 2004; Islam et al., 2004; Ann et al., 2008; Donahoo and Lamour, 2008; Glosier et al., 2008; French-Monar et al., 2009; Sheu et al., 2009; Dunn et al., 2010; Gobena et al., 2012a; Yin et al., 2012; Jiang et al., 2015; Barchenger et al., 2017), increasing the probability of sexual reproduction leading to new races and recombinant oospores resulting in persistence across growing seasons.

Where both mating types exist, sexual reproduction is associated with genetic diversity, persistent pathogens, and often an A1:A2 ratio of ∼1:1 (Lamour and Hausbeck, 2001a; Dunn et al., 2010). Additionally, it has been proposed that there is a climatic influence on mating type distribution. In tropical regions, it is not completely necessary for the pathogen to produce oospores to survive dormantly and clonal lineages may persist for years (Hulvey et al., 2011). A predominance of one or the other mating type has been observed in tropical environments (da Costa Ribeiro and Bosland, 2012; Barchenger et al., 2017). In more temperate environments with greater seasonality, low rates of selfing as well as persistent and a more uniform distribution of mating type has been observed (Lamour and Hausbeck, 2001a; Dunn et al., 2010; Hu et al., 2013b; Carlson et al., 2017). Additionally, exceptions in which there are deviations from a 1:1 ratio in mating type have been observed (Glosier et al., 2008; Sun et al., 2008; Sy et al., 2008; Sheu et al., 2009). This is likely due to the rise of particularly virulent clonal lineages within a growing season and may not reflect the full diversity of a population (Lamour and Hausbeck, 2001b).

### Mutation and Loss of Heterozygosity

The high level of diversity found in *P. capsici* in a single field has also been attributed to mutation and loss of heterozygosity (Lamour et al., 2012; Hu et al., 2013a; Dunn et al., 2014a). Although mutations are the primary source of new genetic variation in oomycetes (Goodwin, 1997), these mutations often cause no observable changes (Silvar et al., 2006). However, rapid genetic changes due to mutation at virulence loci have been observed in *P*. *infestans* and *P*. *sojae* (Drenth et al., 1994, 1996; Förster et al., 1994; Goodwin et al., 1995; Sujkowski et al., 1996).

Recent work with *P*. *capsici* in Taiwan and the closely related species *P*. *colocasiae* (host specific to taro [*Colocasia esculenta* (L.) Schott]), recovered from Nepal, Vietnam, China, and Hawaii, are shedding new light on a novel component to diversity with *P*. *capsici* and *P*. *colocasiae* and very likely, the genus as a whole (Barchenger et al., 2017; Shrestha et al., 2017). Loss of heterozygosity was described in detail in the paper presenting the draft reference genome for *P. capsici* (Lamour et al., 2012). This phenomenon occurred on a large scale, across a high percentage of sexual progeny produced to make a detailed genetic map, and was not specific to any one region of the genome and in total – impacted more than 30% of the *P*. *capsici* genome (Lamour et al., 2012). How it occurs is a mystery but newer sequencing technologies, particularly whole genome sequencing and targeted amplicon sequencing indicate the genomes for *P*. *capsici* and *P*. *tropicalis* can differ dramatically from the diploid state. The difference is not limited to a situation where all the chromosomes are triploid or some other ploidy level, instead it was found that individual isolates can be a mosaic of aneuploid variation. Current work investigating single zoospore progeny from multiple field isolates indicates chromosome dosage can be highly variable within a single zoospore-derived isolate and there is little fidelity to the chromosomal complement of the parental strain. This has potentially significant implications for rapid evolution where gene dosage may allow an isolate to overcome a novel human-mediated selection pressure, including resistance genes and *Phytophthora*-toxic chemicals, and clearly can play a role in the rapid evolution of populations to novel resistance incorporated by the plant breeder.

### Breeding Approaches

Classical breeding approaches for transferring resistance to *P*. *capsici* into adapted chile pepper germplasm has been a goal of many breeding programs. One major challenge to chile pepper breeders is that different inheritance models have been reported among the sources of resistance to *P*. *capsici*. Several laboratories studying CM334 report at least two genes; but often more genes confer resistance (Guerrero-Moreno and Laborde, 1980; Ortega et al., 1991, 1992; Reifschneider et al., 1992; Walker and Bosland, 1999; Thabuis et al., 2003; Sy et al., 2005). Other studies report a single dominant gene (Saini and Sharma, 1978; Kim and Hur, 1990) or a single dominant gene with modifying genes (Barksdale et al., 1984) control resistance such as in PI 201234 and bell pepper (Smith et al., 1967). Multiple genes with additive or epistatic effects are involved in resistance in ‘Perennial’ (Lefebvre and Palloix, 1996). However, it is likely that the qualitative gene model reported for resistance in chile pepper is actually race-specific resistance (Sy et al., 2008; Foster and Hausbeck, 2010) as well as syndrome-specific resistance (Sy et al., 2005). Another effect confounding inheritance studies is variation in the screening techniques among the studies, leading to different interpretations of potentially the same results.

Resistance in chile pepper has polygenetic inheritance based on multimodal distributions and higher order epistasis effects (Pochard and Daubeze, 1980; Palloix et al., 1988; Bartual et al., 1991, 1993; Pflieger et al., 2001; Lefebvre et al., 2002; Ogundiwin et al., 2005; Bonnet et al., 2007; Minamiyama et al., 2007; Truong et al., 2012; Curtis, 2014). Efforts have been made to identify quantitative trait loci (QTL) linked with *P*. *capsici* resistance and transfer these QTLs into elite material (Thabuis et al., 2003, 2004b; Ogundiwin et al., 2005; Sugita et al., 2006; Jin et al., 2007; Minamiyama et al., 2007; Kim et al., 2008; Truong et al., 2012; Liu et al., 2014; Naegele et al., 2014). Although, these QTLs are also often associated with race-specific resistance.

Several molecular markers associated with resistance to *P*. *capsici* have been reported in chile pepper for more rapid selection (Quirin et al., 2005; Kim et al., 2008; Truong et al., 2012; Chomkaeo et al., 2014; Liu et al., 2014; Wang et al., 2016; Xu et al., 2016). However, to date, these publically available molecular markers are generally not widely applicable, and some level of phenotype and genotype mismatch has been observed when they are used in diverse germplasm. This phenotype–genotype mismatch limits selection efficiency for marker assisted selection and also further highlights the high level of plasticity in the pathogen.

Historically, it is difficult to introduce *P*. *capsici* resistance into well-adapted susceptible cultivars. When using classic backcross methods, resistance is lower than the donor parent with threshold effects, which is likely due to the loss of secondary resistance genes (Palloix et al., 1990). Recurrent selection has been used to move polygenic resistance into elite material (Thabuis et al., 2004a). However, linkage drag associated with low yield, small and undesirable fruit, and less vigorous plants is a major limitation to wide adoption of resistant cultivars. Growers would rather plant high yielding, high quality, more uniform cultivars that are susceptible to *P*. *capsici* and risk losing a portion of their crop, than plant less adapted but resistant cultivars. Even cultivars that had field resistance to *P*. *capsici*, e.g., Paladin (Dunn et al., 2014b), became susceptible within a decade as the pathogen evolved new virulence in New Jersey (Krasnow et al., 2017). An excellent example of the boom-and-bust cycle of disease resistance.

Additionally, Reeves et al. (2013) identified an inhibitor to *P*. *capsici* resistance gene (*Ipcr*) in New Mexico Capsicum Accession 10399 (NMCA10399). Their results indicate that a single dominant gene inhibited polygenic host resistance to multiple isolates of *P*. *capsici*. The single dominant gene inhibited resistance to all disease syndromes. The genetic mechanisms of the *Ipcr* gene is unknown; however, it is hypothesized to interfere with upstream recognition sites in the host. Additionally, the frequency of the *Ipcr* gene in commercial cultivars is not known. The authors proved that a chile pepper can be susceptible to *P*. *capsici* for two reasons: lack of *R* genes or presence of an inhibitor gene. These findings further complicate a difficult pathosystem and highlight the complexity of breeding for resistance to *P*. *capsici*.

### Transgenic Issues

Genetic engineering using *Agrobacterium*-mediated transgenic approaches has long been used to increased plant resistance to biotic stresses. Members of Solanaceae such as eggplant, petunia (*Petunia* x *hybrid* Juss.), potato, tobacco (*Nicotiana tabacum* L.), and tomato are readily transformed and are considered model organisms for this technology. However, chile pepper is extremely recalcitrant to *in vitro* regeneration and genetic transformation (Li et al., 2003). Regeneration and transformation of chile pepper has been widely reported (Wang et al., 1991; Zhou et al., 1991; Dong et al., 1992; Lee et al., 1993; Ye et al., 1993; Fari and Andrasfalvy, 1994; Zhu et al., 1996; Jayashankar et al., 1997; Kim et al., 1997; Subhash and Christopher, 1997; Manoharan et al., 1998; Steinitz et al., 1999; Wolf et al., 2001; Li et al., 2003; Lopez-Puc et al., 2006; Arcos-Ortega et al., 2010). However, the problem is an overall lack of reproducibility in these published techniques. One reason for the lack of reproducibility is that regeneration and transformation techniques in chile pepper are genotype-specific (Manoharan et al., 1998). Therefore, different protocols are required depending on the accession being transformed. Furthermore, successfully introduced transgenes in plants regenerated *in vitro* are often not inherited through subsequent generations of self- or cross-pollination. It is hypothesized the transgenes are quickly lost via transposition. More than 81% of the *Capsicum* genome consists of transposons, which is high compared to closely related tomato (50%) and potato (47%) (Qin et al., 2014).

## Strategies

Despite decades of research to better understand the *Capsicum*–*P*. *capsici* pathosystem, *P*. *capsici* is still a major limiting factor for pepper production. Some of the strategies developed in other pathosystems with efficacy in limiting losses associated with infection and disease are not practical for *P*. *capsici*. There is a need to identify strategies that can be adopted to better breed for resistance to *P*. *capsici* in chile pepper.

### Screening Methodology

Breeding for resistance to *P*. *capsici* is heavily dependent on the accuracy and precision of the disease screening method used (Chavez and Kabelka, 2009). Several disease screens have been developed for *P*. *capsici*. For foliar blight screening, using 1,000 zoospores per plant (Alcantara and Bosland, 1994) and 2,000 zoospores per plant using soaked germination paper (Monroy-Barbosa and Bosland, 2010) have been proposed. Additionally, a foliar spray using inoculum has been used. For root-rot screening, 10,000 zoospores per plant (Bosland and Lindsey, 1991) and 100,000 zoospores per plant (Black, 1999) have been used. In addition, a dose of 5,000 zoospores per plant has been used for screening fruit-rot/blight resistance (Biles et al., 1995). Inoculum concentration and plant age play a major role in the level of resistance displayed in the host (da Costa Ribeiro and Bosland, 2012; Barchenger, 2017; Mansfeld et al., 2017). In order to effectively breed for resistance and correctly identify races of *P*. *capsici*, standardized screening protocols should be developed and followed by scientists worldwide.

### Race Nomenclature

As described above, several studies identified physiological races of *P*. *capsici* (Black, 1999; Oelke et al., 2003; Glosier et al., 2008; Monroy-Barbosa and Bosland, 2008, 2010, 2011; Sy et al., 2008; Lee et al., 2010; da Costa Ribeiro and Bosland, 2012; Jiang et al., 2015). Unfortunately, there is a lack of consistency that can limit overall progress among breeders. Some studies use a numerical or alphabetical nomenclature system with the first race designated Race 1 or A (Glosier et al., 2008; Lee et al., 2010). Other studies number races based on virulence with Race 1 being either the most virulent (Sy et al., 2008) or the least virulent (Black, 1999). Despite the inconsistencies in how the races are identified, the most important limitation is they do not provide a naming scheme that allows for more or less virulent races to be described (Barchenger et al., 2017, 2018). Furthermore, there are overlapping names for genetically divergent races.

Over the years many different races for the different disease syndromes of *P*. *capsici* have been identified around the world, and the systems used to designate the different races have no real biological meaning. Therefore, the term race is now being supplemented by a new term, virulence phenotype (Barchenger et al., 2018). Virulence phenotype is used to designate the virulence of the *P*. *capsici* isolate on the various host resistance genes. Races are identified based on the differential reaction with the NMRIL, which defines isolates by resistance genes and will hopefully contribute to practical advances in breeding.

### Global Strategies for Local Gene Deployment

Based on the current knowledge of this complex pathosystem, it may not be possible to develop cultivars with global or even country-wide durable resistance. However, we propose plant breeders utilize global strategies for local gene deployment for *P*. *capsici* resistance. The NMRILs have been used globally (Brazil, China, Taiwan, and across the United States) to characterize *P*. *capsici* for the past decade (Monroy-Barbosa and Bosland, 2008, 2010, 2011; Sy et al., 2008; da Costa Ribeiro and Bosland, 2012; Hu et al., 2013b; Naegele et al., 2014, 2017; Naegele and Hausbeck, 2014; Rehrig et al., 2014; Jiang et al., 2015; Barchenger et al., 2018). The NMRILs provide a host differential to identify the virulence phenotype in a given region at a particular time (Barchenger et al., 2018). Simultaneously, the NMRILs also provide insights into the resistance gene(s) required in that region.

A recommended strategy to breed for resistance in a particular region is to utilize the NMRILs to identify the virulence phenotypes in a given region and compare these to the virulence phenotypes from other regions (Sy et al., 2008; da Costa Ribeiro and Bosland, 2012; Jiang et al., 2015; Barchenger et al., 2018). The resistant NMRILs can then be utilized to move resistance into elite germplasm for region-targeted resistant cultivars. The NMRILs provide information on both the pathogen and the host that can be utilized in developing a resistance breeding strategy in a particular region. This was recently demonstrated in Taiwan (Barchenger et al., 2017), where we conducted targeted sequencing on *P*. *capsici* isolates collected in Taiwan and analyzed the data in terms of the virulence phenotypes developed based on the NMRILs. A clear relationship between polyploidy in the pathogen and fewer susceptible reactions was found among a set of NMRILs. Polyploid isolates were largely present on the East coast of the island and diploid isolates were largely on the West coast, enabling local gene deployment. Utilizing a globally standardized system to characterize resistance on a local scale also allows plant breeders to compare resistance globally and select lines from different countries or regions with similar virulence phenotypes for use in their own breeding program.

### Gene Targeted Resistance

The gene-for-gene model (Flor, 1955, 1971) specifies that in race-specific interactions, the host plant inhibits infection through deployment of defense functions via recognition. This is made possible by the presence of dominant resistance genes in the host that enable recognition of effectors in the pathogen. These effectors encode Pathogen Associated Molecular Patterns/Microbial Associated Molecular Patterns (PAMPs/MAMPs) that are recognized by resistant hosts and trigger Pattern Triggered Immunity (PTI). Successful pathogens, such as *P*. *capsici*, have evolved a large and diverse set of secreted effectors that can suppress PTI and initiate Effector-Triggered Susceptibility (ETS) (Jones and Dangl, 2006; Hein et al., 2009; Gill et al., 2015).

Using the *P*. *capsici* reference genome, Stam et al. (2013) identified pathogen effector proteins. Several effectors produced by *P*. *capsici*, such as those in the RXLR, *Crn*, and PcNpp classes are thought to play important roles in infection of chile pepper. More than 400 candidate RXLP effectors have been identified in the *P*. *capsici* genome (Lamour et al., 2012; Stam et al., 2013). Several necrosis-inducing proteins (*PcNLP*) have been found to play important roles in symptom development in chile pepper (Feng et al., 2011, 2014). Fu et al. (2015) identified several cell-death-inducting members of the pectate lyase gene family (*PcPL*) that were highly induced during infection, and could be effectors. The ethylene-responsive factor CaPTI1 appears to be involved in defense response to *P*. *capsici* (Jin et al., 2015). A single effector, a PcAvr3a-like protein, has been correlated to non-host resistance in several *Nicotiana* species (Vega-Arreguín et al., 2014). Interestingly, Vega-Arreguín et al. (2017) found the non-host resistance mechanisms to *P*. *capsici* are the same as the mechanism for host-resistance. Selections made within the landrace CM334 act like a non-hosts because no isolates, to date, can infect. To be useful for chile pepper breeding, the effector targets in host differentials derived from CM334 need to be identified. The resistant parent of the host differential NMRILs, CM334, has been sequenced (Kim et al., 2014), which is an important step in identifying effector targets. However, to date, no efforts have been made to identify these regions in the NMRILs.

Although detection of effector targets in the host are limited, efforts have been made to identify resistance genes (Silvar et al., 2008; Wang J.E. et al., 2013; Zhang et al., 2013; Rehrig et al., 2014; Xu et al., 2016). Mallard et al. (2013) identified a major QTL, *Pc5.1*, located on chromosome 5 associated with resistance to 12 isolates of *P*. *capsici* from different geographic regions. It has been widely reported that *P*. *capsici* resistance genes are clustered on chromosome 5 (Bonnet et al., 2007; Truong et al., 2012; Liu et al., 2014; Rehrig et al., 2014; Wang et al., 2016). The authors conducted a meta-analysis and found this QTL is highly conserved among diverse resistant chile pepper accessions. Several resistance genes are within and very near to *Pc5.1*, including *CaPhyto* (Wang et al., 2016), *CaDMR1* (Rehrig et al., 2014) and likely others (Liu et al., 2014). The *C*. *annuum* Polygalacturonase-inhibiting Protein1 (*CaPGIP1*) gene has been identified as to reduce susceptibility in GM tobacco plants (Wang X. et al., 2013). The *PGIPs* are extracellular plant proteins with recognition ability against many PGs produced by fungi (De Lorenzo et al., 2001). Furthermore, *ChiIV3* is a positive regulator of plant cell death and triggers defense signaling and upregulation of pathogenesis related genes against *P*. *capsici* infection (Liu et al., 2017). Interestingly, there appears to be several different types of *R* genes in *Capsicum*. The majority of the *R* genes are nucleotide-binding and leucine-rich-repeat proteins (NLRs). Work in *R* gene identification is more extensive in other Solanaceae crops, and several NLRs have been identified with high orthology to those in tomato and potato (Kim et al., 2014). Recent findings suggest massive expansion of NLR genes in *Capsicum*, largely due to long-terminal-repeat-retrotransposons-mediated retroduplication (Kim et al., 2017). Richins et al. (2010) identified 168 differentially expressed genes under root-rot inoculation of *P*. *capsici* and one of these genes, *XEGIP*, was further characterized by Jones et al. (2015). The *XEGIP* gene is modeled to inhibit xyloglucan-specific endo β-1,4 glucanase produced by *P*. *capsici* and attacks the xyloglucan bonds in plant cell walls (Yoshizawa et al., 2012). However, the capacity of these genes to recognize PAMPs is unknown. Despite the high number and diversity of resistance genes, breeding for resistance to *P*. *capsici* is still inadequate and other strategies are needed to more effectively develop durable resistant cultivars. Further genome-wide analysis of the evolution of NLRs and effectors could provide a basis for gene-targeted resistance breeding.

A potential challenge to identifying effector targets in this host is the presence of the *Icpr* gene as accessions containing the *Icpr* gene are always completely susceptible (Reeves et al., 2013). The mode of action and the frequency of the *Icpr* gene in *Capsicum* populations are unknown.

### Double Haploid Development

As previously described, the use of durable sources of *P. capsici* resistance in traditional chile pepper breeding programs has limitations. However, the use of double haploid (DH) technology could be a way to fix resistance genes in elite material. Hybridizing adapted material with good horticultural traits to accessions with high levels of resistance and developing DH lines from the F_1_ generation will allow plant breeders to quickly fix resistance without losing the important horticultural traits. However, there are major limitations to using DH lines, including high cost, necessity of expertise in tissue culture, and the development of protocols specific to each laboratory and genotype. Double haploids have been developed in chile pepper in the past; however, most of the time, success rates are generally low (Dumas de Vaulx et al., 1981; Vagera and Havranek, 1985; Morrison et al., 1986; Munyon et al., 1989; Kristiansen and Andersen, 1993; Maheswary and Mak, 1993; Qin and Rotino, 1993; Ltifi and Wenzel, 1994; Mitykó et al., 1995; Dogimont et al., 1996; Dolcet-Sanjuan et al., 1997; Gyulai et al., 2000; Supena et al., 2006). The reason for the high failure rate is unknown, but it is well known that *Capsicum* is highly recalcitrant to *in vitro* regeneration and the media required is highly genotype specific.

### Omnigenics

Resistance to *P*. *capsici* in chile pepper is a highly complex trait. Through genome-wide association studies, the understanding of the genetic basis of complex traits has greatly expanded. Many important loci generally have small effects and complex traits are largely influenced by non-coding variants such as promoters or enhancers (Boyle et al., 2017). Common SNPs distributed throughout the genome with effects below detectable significance levels account for a large portion of the heritability of complex traits (Yang et al., 2010). Therefore, Boyle et al. (2017) proposed the omnigenic model which postulates that most heritability can be explained by effects on genes outside core disease-related pathways. They suggest that essentially any gene with regulatory variants in at least one tissue that contributes to pathogenicity is likely to have non-trivial effects on disease resistance.

While genes conferring resistance in chile pepper have largely been localized on chromosome 5 (Mallard et al., 2013; Kim et al., 2017), no loci have been identified that account for resistance over a wide geographical region or in diverse genetic backgrounds. It is likely that a large number of variants contribute to resistance (Kim et al., 2017). Therefore, the omnigenic model suggests that to understand the whole picture of disease resistance, we should not only study core genes and pathways, but also the multitude of variants throughout the genome that have seemingly small effects on resistance. The omnigenic model has the potential explain why developing molecular markers and breeding for resistance to *P*. *capsici* in chile pepper has been limited in the past and should be considered when breeding for resistance in the future.

### FAST SNP Markers for Increased Selection Accuracy

The prediction accuracy from genomic selection for most crop species is generally between 40 and 65%, and essentially never 100% (as reviewed by Fu et al., 2017), and this is also true for most molecular markers available for *P*. *capsici* resistance in chile pepper. Therefore, Fu et al. (2017) proposed the development of function-associated specific trait (FAST) SNP markers, as an alternative to regular genome selection, for rapid and more accurate trait predictions. The FAST SNPs technology and has not been employed on vegetable crops (including chile pepper) with large and complex genomes; however, FAST SNPs may increase *P*. *capsici* resistance prediction accuracy. To facilitate the development of FAST SNP markers, Fu et al. (2017) proposed a procedure based on RNA-seq of 10 or more pairs of individual plants with extreme trait values (resistant vs. susceptible). Ideally, the lines used for FAST SNP marker development are derived from diverse resistance sources. The RNA-seq reads for each pair of lines are then *de novo* assembled and differential transcripts are identified. Trait-specific markers can then be developed based on consensus SNPs among all the pairs. FAST SNP markers have the potential to offer better marker-based trait prediction; however, more empirical investigations are needed to confirm their true value. Due to the large genome size of the host, the plasticity of the pathogen genome, and the lack of widely applicable molecular markers makes the multifaceted *Capsicum*–*P*. *capsici* pathosystem an attractive candidate for FAST SNP validation in vegetables.

## Conclusion

These are exciting times for plant and pathogen research as new tools, particularly at the genomic level, become available and more affordable. Combinations of strategies and collaborative efforts from scientists around the world are required to effectively breed for resistance to *P*. *capsici*. Progress in understanding and manipulating the *Capsicum*–*P*. *capsici* system is likely to be useful in other complex host–pathogen systems and increase our odds to develop durable management strategies.

## Author Contributions

All authors listed have made a substantial, direct and intellectual contribution to the work, and approved it for publication.

## Conflict of Interest Statement

The authors declare that the research was conducted in the absence of any commercial or financial relationships that could be construed as a potential conflict of interest.
